# Micronutrient Status in Pregnant Women after Metabolic Bariatric Surgery in the United Arab Emirates: A Prospective Study

**DOI:** 10.3390/nu16010072

**Published:** 2023-12-25

**Authors:** Amna Al Mansoori, Mo’ath F. Bataineh, Hazem Al Momani, Habiba I. Ali

**Affiliations:** 1 Department of Nutrition and Health, College of Medicine and Health Sciences, United Arab Emirates University, Al Ain P.O. Box 15551, United Arab Emirates; 201790103@uaeu.ac.ae (A.A.M.); m.bataineh@uaeu.ac.ae (M.F.B.); 2 Weight Management Unit, NMC Royal Khalifa Hospital, Abu Dhabi P.O. Box 35233, United Arab Emirates; almomanih@yahoo.co.uk

**Keywords:** bariatric surgery, pregnancy, vitamin B_12_, vitamin D, iron

## Abstract

Metabolic bariatric surgery (MBS) helps reduce comorbidities, such as hypertension and gestational diabetes, and is more effective than diet management for women with obesity-related health issues. Vitamin B_12_, vitamin D, and iron play important roles in ensuring the health of a neonate. However, pregnancies occurring after MBS may face complications related to micronutrient deficiencies, particularly of vitamins B_12_ and D and iron. This study aimed to investigate the vitamin B_12_, vitamin D, ferritin, and iron status of pregnant women who underwent MBS compared with women without MBS history. The study included 217 pregnant women (105 with a history of MBS and 112 without a history of MBS) who visited a major maternity hospital in Abu Dhabi, United Arab Emirates (UAE) between July 2021 and November 2022. The maternal vitamin B_12_, vitamin D, iron, and ferritin levels were measured twice, initially during the first or second trimester and subsequently during the third trimester. The iron was measured once during the pregnancy. Vitamin B_12_ deficiency was higher among pregnant women with MBS history compared to non-bariatric pregnant women (24.4% vs. 3.9%, *p* < 0.001). Women with a history of MBS had a higher prevalence of vitamin D deficiency (62.3% vs. 37.7%, *p* < 0.002). Linear regression analysis indicated that vitamin B_12_ levels decreased by 55 pg/mL in women with a history of MBS and by 4.6 pg/mL with a unit increase in body mass index (kg/m^2^). Furthermore, vitamin D levels in women with a history of MBS decreased by 4.9 ng/mL during pregnancy. Metabolic bariatric surgery is associated with vitamin B_12_, vitamin D, and iron deficiencies during pregnancy.

## 1. Introduction

Pre-pregnancy obesity is a risk factor for various maternal and fetal outcomes, such as labor induction, miscarriage, and gestational diabetes [1,2]. Different interventions, such as diet, exercise, and medications, fail to manage the weight of individuals with obesity (body mass index [BMI] ≥ 40 kg/m^2^) [3,4]. Hence, metabolic bariatric surgery (MBS) is increasingly used to treat morbidities in individuals with obesity.

There is a well-established association between the perinatal environment and potential future disease [5,6,7,8,9,10,11]. Adequate maternal nutrition is crucial for fetal growth and development [12,13]. It supports the physiological demands of the body to support the placenta and offspring during pregnancy [12]. Pregnant women with a history of MBS have been found to experience micronutrient deficiencies [14,15,16,17,18,19], with the most common being vitamin B_12_ and D deficiencies [20]. A recent systematic review revealed the association between bariatric surgery and pregnancy complications such as low gestational weight gain, which affects neonatal weight [21]. Vitamin B_12_ and folic acid are crucial for the development of the fetal central nervous system and are involved in the methylation and decarboxylation processes necessary for DNA and catecholamine neurotransmitter synthesis [22,23,24]. Vitamin D also plays a vital role in maintaining a healthy pregnancy and plays an anti-inflammatory role in pregnancy, regulating placental immunity and stimulating antimicrobial responses [18]. Maternal iron deficiency can lead to fetal hypoxia during a high-demand period [25] and may also lead to long-term neurobehavioral abnormalities and cardiovascular diseases [26]. Ferritin and hemoglobin levels normally decrease during pregnancy [27], which is further exacerbated in women with a history of MBS. The surgery-to-pregnancy time interval may affect micronutrient levels during pregnancy [28].

The Arab Gulf region, despite having one of the highest rates of MBS, has comparatively fewer research outcomes in this field than its Western counterparts [29]. Furthermore, although obstetricians in the UAE are encountering increasing numbers of pregnant women with prior metabolic bariatric surgery, there is a lack of data on micronutrient status and maternal perinatal outcomes in this cohort of women. In addition, there are no uniform micronutrient screening guidelines for pregnant women with previous MBS in the UAE. Therefore, the main research question this study was designed to address was to what extent previous MBS can affect the micronutrient levels of pregnant women in the UAE. The objectives of the study were to (1) assess vitamin B_12_, vitamin D, and iron levels in pregnant women with a history of MBS and (2) assess the impact of the type of MBS and the surgery–pregnancy time interval (STP) on the maternal status of vitamin B_12_, vitamin D, and iron during pregnancy. It was hypothesized that that pregnant women with a history of MBS will have lower vitamin B_12_, vitamin D, and iron statuses compared with those without prior MBS.

## 2. Materials and Methods

### 2.1. Study Design

This prospective study enrolled pregnant women with a history of MBS and a control group comprising pregnant women without a history of MBS. Women were recruited from their first booking and followed during pregnancy until delivery. The primary outcomes were vitamin B_12_, vitamin D, and iron statuses in pregnant women. This study was conducted between July 2021 and October 2022.

### 2.2. Setting and Participants

The recruitment site of the participants in this study was one of the major maternity hospitals in Abu Dhabi, UAE, which offers prenatal care services to pregnant women with previous MBS, such as intramuscular B_12_ and iron infusions, when needed. The pregnant women attending the hospital serving as the recruitment site came from different regions (emirates) of the UAE. 

The inclusion criteria were pregnant women with singleton aged 18–45 years with a gestational age of ≤28 weeks. Women who had previously undergone sleeve gastrectomy (SG) or Roux-en-Y gastric bypass (RYGB) surgery were eligible for recruitment. Women who had undergone other types of MBS or those who had undergone MBS more than once were excluded. 

### 2.3. Data Collection

The medical records of the participants were used to obtain information, such as nationality, education level, smoking status, having in vitro fertilization (IVF), delivery type, twin pregnancy, MBS type, surgery-to-pregnancy interval (months), history of vitamin B_12_ intramuscular (IM) injections, history of iron infusion, and vitamin–mineral supplement intake. Pregnancy-related complications, such as gestational diabetes, hypertension, and hypothyroidism were also extracted from the medical records. 

Vitamin B_12_ and D levels were measured twice during the pregnancy during the 1st or 2nd trimester and subsequently during the 3rd trimester. Iron was measured once at the booking time before 28 gestational weeks. Blood was collected from the cephalic or median cubital vein into vacutainer serum-separating tubes (Becton Dickinson and Company, Franklin Lakes, NJ, USA) by a certified phlebotomist at the hospital. Biochemical tests were performed according to the laboratory reference of the hospital. Parameters such as vitamin B_12_, vitamin D, ferritin, iron, and hemoglobin levels were analyzed using the Alinity H-series Integrated Hematology System (Abbott, Chicago, IL, USA) with the help of reagent kits and quality controls from the manufacturer. The normal range provided by the kit manufacturer was used as a reference. Vitamin B_12_ levels were measured using a chemiluminescent microparticle intrinsic factor assay. Vitamin D and ferritin levels were measured using a chemiluminescent microparticle immunoassay. The iron levels were measured using direct calorimetric determination without deproteinization.

### 2.4. Sample Size

The sample size was calculated using G*Power software (version 3.1.9.7) to compare the two groups (MBS vs. non-MBS). The analysis suggested a minimum sample size of 210 participants (a minimum of 105 participants in each group) to detect two-tailed significance with a medium effect size (0.5), an alpha equal to 0.05, and a power of 0.95. Accordingly, 217 pregnant women were recruited for the study.

### 2.5. Ethics Approval

The ethical approval of the study was obtained from the Research and Ethics Committee of the hospital (No. RP DAE/2021/102) on 15 June 2021. Written informed consent was obtained from all participants prior to their enrollment in the study.

### 2.6. Statistical Analysis

All data were analyzed using IBM SPSS Statistics, version 28 (IBM Corp., Armonk, NY, USA). The assumption of data normality for the entire study was tested using the Kolmogorov–Smirnov test. For continuous variables, parametric tests were used when the normality assumption was satisfied, and data were reported as mean ± standard deviation (SD). When the normality assumption was not satisfied, non-parametric tests were used, and the data were reported as median and interquartile ranges. Categorical variables are presented as frequencies and percentages. An independent *t*-test was used to compare normally distributed continuous variables, and the Mann–Whitney U test was used for non-normally distributed continuous variables. Categorical variables were tested using the chi-square test. Univariate analysis was used to assess the correlation between variables; variables with a *p* < 0.2 were subsequently included in a multiple regression model. Multiple linear regression was used to assess predictors of maternal vitamin B_12_ levels.

## 3. Results

**Figure 1:**
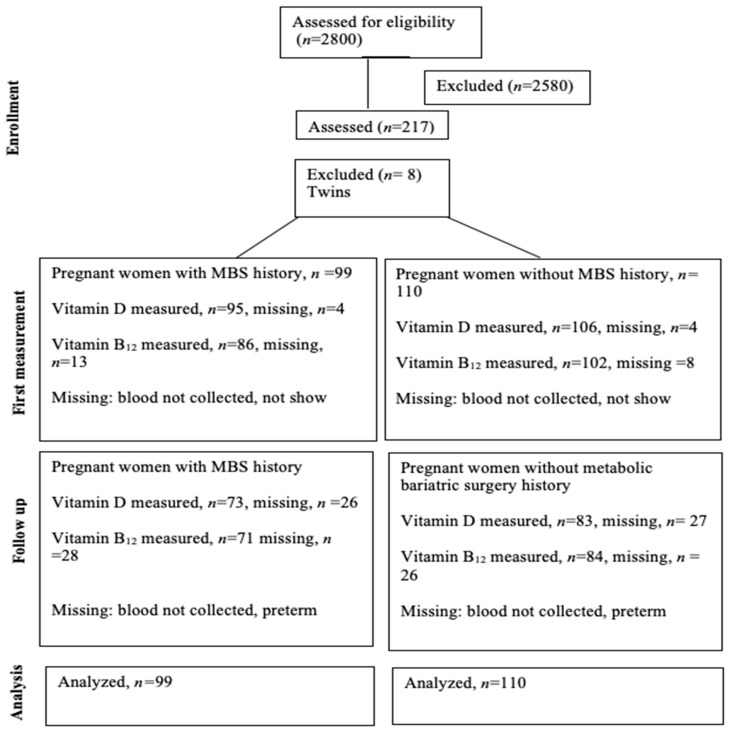
Flow of the participants in the study.

Table 1 summarizes the sociodemographic characteristics of the 106 pregnant women who underwent MBS and the 111 pregnant women who did not. The flow chart of the participants in the study is shown in Figure 1. The participants of this study were from different regions of the UAE (Supplementary Table S1). Women with a history of MBS were older and had a higher pre-pregnancy BMI than those without a history of MBS (*p* < 0.001). Employment status differed significantly between women with and without a history of MBS (*p* < 0.040). The incidence of twin pregnancies was higher among women with a history of MBS than in those without a history of MBS (*p* < 0.017).

Table 2 shows that women with MBS history had significantly lower levels of vitamin B_12_ and D (*p* < 0.001) and mean hemoglobin (*p* < 0.004) than those without MBS history in the first and second trimesters. Vitamin B_12_ levels were significantly reduced further during the third trimester in women with an MBS history than in those without an MBS history (*p* < 0.034). A significantly higher proportion of women with MBS history had vitamin B_12_ deficiencies (*p* < 0.001). Vitamin D deficiency was significantly higher in women with MBS history than in those without MBS history (*p* = 0.002) in the first and second trimesters. However, Vitamin D increased significantly in the third trimester in the MBS group compared to the non-MBS group (*p* < 0.001). Hemoglobin levels were higher in women with MBS history than in those without MBS history (*p <* 0.004). Women with a history of MBS showed a significantly greater requirement for iron infusion than those without a history of MBS (*p* < 0.001). The administration rate of vitamin B_12_ via IM injection was five times higher in the MBS cohort than in the non-MBS cohort (*p* < 0.001). The twin cases were excluded (two from non-MBS and six from MBS). Therefore, there were 99 pregnant women with MBS history and 110 women without MBS history.

The impact of MBS type on micronutrient status is shown in Table 3. There was no significant difference between the groups.

Table 4 indicates that no significant differences in micronutrient status were observed between the women who conceived 18 months or less after the surgery (early pregnancy) and those with a surgery-to-pregnancy interval of more than 18 months (late pregnancy). However, a higher proportion of women who conceived after 18 months received iron infusions compared to those who conceived within 18 months after surgery (*p* < 0.031).

Table 5 shows the supplement intake of the participants during pregnancy. There was a significant difference in oral iron (*p* < 0.03). Only a minority of pregnant women with MBS received vitamin supplements.

## 4. Discussion

This prospective study investigated the status of vitamins B_12_ and D, iron, and ferritin in pregnant women with and without a history of metabolic bariatric surgery, with a comparison cohort of pregnant women without metabolic bariatric surgery history in the UAE, considering the surgery type as well as the pregnancy-to-surgery time interval. The current study showed that deficiencies in vitamins B_12_ and D and iron are associated with metabolic bariatric surgery. Vitamin B_12_ deficiency was higher in women with MBS history than in those without MBS history, despite the supplementation (24.4% vs. 3.9%) in the first and second trimesters. MBS was associated with vitamin B_12_ deficiency due to alterations in stomach acidity, which is important for vitamin B_12_ absorption and the absence or reduced secretion of intrinsic factors. IM injections of vitamin B^12^ are recommended by the British Obesity and Metabolic Society, particularly for those with persistent deficiency, despite receiving high oral doses of vitamin B_12_ [30]. 

The absence of early intervention can explain this finding; only 32% of patients received vitamin B_12_ IM injections. The higher prevalence of vitamin B_12_ deficiency in the MBS group was comparable to that reported in previous studies [20,27,31,32,33,34].

A reduction in vitamin B_12_ levels during pregnancy, from 309 pg/mL in the first and second trimesters to 254 pg/mL in the third trimester, was detected in pregnant women without MBS history. This can be explained by the expansion of body volume and higher excretion of water-soluble vitamins, including vitamin B_12_, in the urine [35]. This finding is consistent with that of a previous study [36], which showed that vitamin B_12_ levels decreased gradually from the first trimester to the third trimester. The present study also showed that the prevalence of vitamin D deficiency was higher among women who underwent MBS than in those who did not at the booking time (62.3% vs. 37.7%, *p* = 0.002). This might be due to lipid malabsorption and the restriction of certain foods, such as dairy, due to lactose intolerance, particularly in the RYGB cohort [37,38]. This finding is consistent with those of other previous studies [27,39] that reported a higher deficiency of vitamin D among women with MBS history. 

Low vitamin D among women with MBS history compared to non-MBS women at the beginning of pregnancy could be attributed to the higher BMI among MBS women compared with non-MBS women (28.74 kg/m^2^ vs. 25.28 kg/m^2^, *p* < 0.001). A meta-analysis [40] showed that higher BMI was associated with vitamin D deficiency. An improvement in vitamin D status during pregnancy during the third trimester was detected in the MBS cohort in this study. This was attributed to vitamin D supplementation adherence and was consistent with the findings of Devlieger et al. [27], who reported a reduction in vitamin D deficiency, from 14% to 6%, before delivery due to vitamin D supplementation.

The current study found a higher rate of iron deficiency anemia in women with an MBS history compared to those without an MBS history. This finding is consistent with the findings of previous research [41,42,43]. Similar findings were reported by a recent meta-analysis [44]. However, in the present study, hemoglobin levels were improved in the MBS cohort compared to the non-MBS cohort. This may be attributed to the higher rate of intravenous iron infusions (67% vs. 33.6%) and vitamin B_12_ injections (21.6% vs. 3.6%) in the MBS cohort than in the non-MBS cohort.

Regarding surgery types, we did not find statistically significant differences in vitamin deficiency. In contrast, previous research [22,36,39] found that deficiencies of vitamins B_12_ and D were higher in RYGB cohorts. Bypassing of the sites of vitamin B_12_ absorption in the RYGB cohort may explain the higher deficiency in the RYGB cohort. However, we found higher IDA in the SG cohort. This finding is consistent with that of a previous study [45], which indicated a higher incidence of anemia in an SG cohort than in an RYGB cohort at the 2-year follow-up. The greater need for iron infusions in the SG cohort than in the RYGB cohort in this study can be attributed to the higher IDA rates in the SG cohort.

Women are advised to wait for 18 months post-MBS before conceiving because this weight loss period may carry risks for mothers and neonates [46,47,48]. This study found that 50.5% of women conceived within 18 months of undergoing MBS [49]. However, the surgery-to-pregnancy interval of 18 months or less was not associated with vitamin B_12_ deficiency; although, the incidence of vitamin B_12_ deficiency was slightly higher compared to those who conceived more than 18 months after the surgery (25% vs. 23.8%, respectively). This result was consistent with those reported by previous studies [28,50], which reported higher vitamin B_12_ deficiencies in women with shorter surgery-to-pregnancy intervals. 

In the current study, vitamin D deficiency was higher in the shorter STP interval cohort than in the longer STP interval cohort. This finding contradicts that of a study [28] that showed a higher rate of vitamin D deficiency in the longer STP interval cohort. This may be explained by the different cutoff values used to determine vitamin D deficiency; Doline et al. used a cutoff value of <30 ng/mL, while the present study used 20 ng/mL as the cutoff value.

Regarding STP, this study revealed that iron infusion was often needed. IDA rates were higher in women with longer STP intervals than in those with shorter STP intervals. This finding is consistent with that of previous studies [51,52,53,54] that reported that IDA was higher in women with STP intervals of >18 months. 

The rate of anemia increased after 4 years in the MBS population, which suggests the importance of considering the time interval when studying IDA [55]. In contrast, another study showed that this finding of a higher IDA rate in the longer STP interval cohort is not consistent with the results of [56], which showed an association between early conception of <18 months and IDA rate among the MBS population. These contrasting results may be attributed to the small sample size as this study had only seventy-one MBS subjects [56].

This study revealed a greater need for iron infusion among the late pregnancy cohort compared to the early pregnancy cohort (77.6% vs. 57.1%, *p* < 0.031). This higher rate is attributed to the higher prevalence of iron deficiency anemia among the late cohort. This finding is consistent with that of a retrospective study [56], which showed that the rate of iron infusion in the late cohort was 57%. In the present study, 86% of women with an SG history conceived >18 months after MBS, compared with only 14% of women with an RYGB history. This could explain the higher IDA rate in the SG cohort in this study. This further emphasizes the importance of considering the STP interval when assessing IDA after MBS. The increased risk of micronutrient deficiency, particularly that of vitamin B_12_, in women with a history of RYGB, requires further investigation. 

## 5. Conclusions

More attention should be paid to vitamin B_12_ deficiency in women with MBS history, and an early intervention to manage vitamin B_12_ deficiency should be taken into consideration before and after pregnancy. Moreover, only 32% of participants with a vitamin B_12_ deficiency received vitamin B_12_ supplementation four times (1 mg each). Therefore, it is important to follow up with women with MBS history and ensure that they are on supplements since only a minority of the participants in this study were on supplements. 

Further studies involving larger-scale and multiple centers are recommended. The main limitation of the present study is that it included women who were recruited from a single hospital. However, this hospital is one of the largest obstetric hospitals in the UAE that serves women with previous MBS and represents the whole country. Moreover, the participants in this study were from various regions of the UAE. This study was originally designed to involve two hospitals but, due to the COVID-19 pandemic, recruitment at one of the hospitals was not possible. On the other hand, ours is the only prospective study that compares the micronutrient status of pregnant women with MBS history and those with no MBS history in the UAE. The prospective design used is also a major strength of the present study.

## Figures and Tables

**Table 1:** Demographic characteristics of the enrolled participants (*n* = 217).

Variables	Total Population (*n* = 217)	Metabolic Bariatric Surgery (*n* = 105)	Non-Metabolic Bariatric Surgery (*n* = 112)	*p* Value
Age, years (mean ± SD)	31.47 ± 5.62 years	32.69 ± 5.13 years	30.30 ± 5.84 years	0.001 ^b^
Age group, *n* (%)				0.020 ^a^
≤35 years	155 (71.4)	68 (64.2)	87 (78.4)	
>35 years	62 (28.6)	38 (35.8)	24 (21.6)	
Nationality, *n* (%)				<0.001 ^a^
Non-Emirati	45 (20.7)	9 (8.5)	36 (32.4)	
Emirati	172 (79.3)	97 (91.5)	75 (67.6)	
Educational level, *n* (%)				0.406 ^a^
High school	50 (23)	27 (25.5)	23 (20.7)	
College	167 (77)	79 (74.5)	88 (79.3)	
Working, *n* (%)				0.040 ^a^
No	152 (70.4	67 (63.8)	85 (76.6)	
Yes	64 (29.6)	38 (36.2)	26 (23.4)	
Smoking, *n* (%)				0.689 ^a^
No	212 (97.7)	104 (98.1)	108 (97.3)	
Yes	5 (2.3)	2 (1.9)	3 (2.7)	
Pre-pregnancy BMI median (IQR), kg/m^2^	27.19 (24.22–30.49) kg/m^2^	28.74 (25.53–31.64) kg/m^2^	25.28 (23.71–29.27) kg/m^2^	<0.001 ^c^
IVF, *n* (%)				0.132 ^a^
No	209 (96.3)	100 (95.3)	109 (98.2)	
Yes	8 (3.7)	6 (5.7)	2 (1.8)	
Twins, *n* (%)				0.017 ^a^
No	209 (96.3)	99 (94.3)	110 (98.2)	
Yes	8 (3.7)	6 (5.7)	2 (1.8)	
Hypertension, *n* (%)				0.376 ^a^
No	211 (97.2)	102 (96.2)	109 (98.2)	
Yes	6 (2.8)	4 (3.8)	2 (1.8)	
Gestational diabetes, *n* (%)				0.430 ^a^
No	148 (68.2)	75 (70.8)	73 (65.8)	
Yes	69 (31.8)	31 (29.2)	38 (34.2)	
Hypothyroidism *n*, (%)				0.088 ^a^
No	180 (86.1)	81 (81.8)	99 (90)	
Yes	29 (13.9)	18 (18.2)	11 (10)	

^a^ Chi-square test, ^b^ independent *t*-tests, ^c^ Mann–Whitney U test. SD, standard deviation; IVF, in vitro fertilization. Significance at 5%: Groups were compared using chi-square test. Continuous variables were compared using independent *t*-tests and skewed data were compared using the Mann–Whitney U test.

**Table 2:** Micronutrient status among women with and without MBS history (*n* = 209).

Variables	Reference	Metabolic Bariatric Surgery (*n* = 99)	Non-Metabolic Bariatric Surgery (*n* = 110)	*p* Value
First vitamin B_12_ blood level, median (IQR)	187–883 pg/mL	236 (186–307) pg/mL	309 (242–388) pg/mL	< 0.001 ^c^
Second vitamin B_12_ level, median (IQR)	187–883 pg/mL	221 (195–269) pg/mL	254 (204–333) pg/mL	0.034 ^c^
Vitamin B_12_ deficiency during first blood collection (%)	<187 ng/mL	24.4	3.9	<0.001 ^b^
Vitamin B_12_ deficiency during second blood collection (%)	<187 ng/mL	19.2	15.5	0.453 ^b^
First vitamin D level (mean ± SD)	20–50 ng/mL	22.66 ± 9.86 ng/mL	27.90 ± 12.79 ng/mL	0.001 ^b^
Second vitamin D level (mean ± SD)	20–50 ng/mL	34.62 ± 11.87 ng/mL	28.10 ± 11.28 ng/mL	<0.001 ^b^
Vitamin D deficiency during first blood collection (%)	<20 ng/mL	62.3	37.7	0.002 ^a^
Vitamin D deficiency during second blood collection (%)	<20 ng/mL	23.9	10.8	0.044 ^b^
Hemoglobin level at booking (mean ± SD)	11.6–15 g/dL	10.96 ± 1.13 g/dL	10.49 ±1.21 g/dL	0.004 ^b^
Low hemoglobin (%)	<11.6 g/dL	80.8	66.4	0.021 ^a^
Ferritin level at booking, median (IQR)	4.63–204 ng/mL	7.30 (4.93–12.35)	7.31 (4.87–12.58)	0.856 ^c^
Low ferritin level (%)	<4.63 ng/mL	57	42.3	0.838
Serum iron level (mean ± SD)	20–162 mg/dL	57.47 ± 37.95 mg/dL	46.67 ± 26.08 mg/dL	0.298 ^b^
Iron deficiency anemia, *n* (%)	131(63.6)	65 (67)	66 (60.6)	0.336 ^a^
Iron infusion, *n* (%)	103 (49.5)	66 (67.3)	37 (33.6)	<0.001 ^a^
Vitamin B_12_ injection, *n* (%)	25 (12.1)	21 (21.6)	4 (3.6)	<0.001 ^a^

^a^ Chi-square test, ^b^ independent *t*-tests, ^c^ Mann–Whitney U test. SD, standard deviation. Significance at 5%: Groups were compared using chi-square test. Continuous variables were compared using independent *t*-tests and skewed data were compared using the Mann–Whitney U test.

**Table 3:** Micronutrient status in women with metabolic bariatric surgery history including SG and RYGB (*n* = 99).

Variables	Reference	SG (*n* = 77)	RYGB (*n* = 22)	*p*-Value
First vitamin B_12_ level, median (IQR)	187–883 pg/mL	244 (191.5–330) pg/mL	207 (182–275) pg/mL	0.155 ^c^
Second vitamin B_12_ level, median (IQR)	187–883 pg/mL	221 (198–269) pg/mL	217 (176–352) pg/mL	0.843 ^c^
First vitamin D level (mean ± SD)	20–50 ng/mL	23.21 ± 9.30 ng/mL	20.61 ± 11.75 ng/mL	0.297 ^a^
Second vitamin D level (mean ± SD)	20–50 ng/mL	29.40 ± 10.76 ng/mL	23.89 ± 12.21 ng/mL	0.079 ^a^
Hemoglobin level (mean ± SD)	11.6–15 g/dL	10.57 ± 1.13 g/dL	10.21 ± 1.48 g/dL	0.234 ^a^
Low hemoglobin, *n* (%)	<11.6 g/dL	65 (84.4)	18 (81.8)	0.770 ^a^
Ferritin level, median (IQR)	4.63–204 ng/mL	7.05 (4.73–13) ng/mL	8.24 (5.29–12.63) ng/mL	0.549 ^c^
Low ferritin, *n* (%)	<4.63 ng/mL	14 (26.9)	3 (18.8)	0.509 ^a^
Serum iron level (mean ± SD)	20–162 μg/dL	49.32 ± 27.84 μg/dL	36.60 ± 16.23 μg/dL	0.343 ^a^
Iron infusion, *n* (%)				0.261 ^a^
Yes		54 (71.1)	12 (57.1)	
No		23 (29.9)	9 (42.9)	
Viatmin B_12_ injection, *n*(%)				0.149 ^a^
Yes		14 (18.4)	7 (33.3)	
No		62 (81.6)	14 (66.7)	

^a^ Chi-square test, ^c^ Mann–Whitney U test. SD, standard deviation; SG, sleeve gastrectomy; RYGB, Roux en Y gastric bypass. Significance at 5%: Groups were compared using chi-square test. Skewed data were compared using the Mann–Whitney U test. The first vitamin levels were collected during 1st and 2nd trimesters and the second vitamin levels were collected in 3rd trimester.

**Table 4:** Impact of surgery-to-pregnancy interval on micronutrient status in women with MBS (*n* = 99).

Variables	Reference	≤18 Months (*n* = 49)	>18 Months (*n* = 50)	*p* Value
First vitamin B_12_ blood level, median (IQR)	187–883 pg/mL	233 (185.75–299) pg/mL	237 (186–358) pg/mL	0.966 ^c^
Second vitamin B_12_ level, median (IQR)	187–883 pg/mL	232 (198–325) pg/mL	216 (192–251) pg/mL	0.125 ^c^
First vitamin D level (mean ± SD)	20–50 ng/mL	22.94 ± 10.01 ng/mL	22.40 ± 9.80 ng/mL	0.791 ^b^
Second vitamin D level (mean ± SD)	20–50 ng/mL	27.70 ± 10.91 ng/mL	28.50 ± 11.83 ng/mL	0.771 ^b^
Hemoglobin level (mean ± SD)	11.6–15 g/dL	10.60 ± 1.22 g/dL	10.38 ± 1.20 g/dL	0.364 ^b^
Ferritin level, median (IQR)	4.63–204 ng/mL	7.15 (4.5–11.66) ng/mL	7.56 (4.94–15.95) ng/mL	0.511 ^c^
Ferritin deficiency, *n* (%)	<4.63 ng/mL	27 (55.1)	21 (42)	0.419 ^a^
Serum iron level (mean ± SD)	(20–162) µg/dL	51.38 ± 25.40 µg/dL	44.31 ± 26.92 µg/dL	0.544 ^b^
Iron deficiency anemia, *n* (%)				0.221 ^a^
Yes		30 (61.2)	35 (72.9)	
No		19 (38.8)	13 (27.1)	
Iron infusion, *n* (%)				0.031 ^a^
Yes		28 (57.1)	38 (77.6)	
No		21 (42.9)	11 (22.4)	
Vitamin B_12_ injection, *n* (%)				0.847 ^a^
Yes		11 (22.4)	10 (20.8)	
No		38 (77.6)	38 (79.2)	

^a^ Chi-square test, ^b^ independent *t*-tests, ^c^ Mann–Whitney U test. SD, standard deviation. Significance at 5%: Groups were compared using chi-square test. Continuous variables were compared using independent *t*-tests and skewed data were compared using the Mann–Whitney U test.

**Table 5:** Supplement intake of the participant during pregnancy.

Variables	Total Population (*n* = 209)	Metabolic Bariatric Surgery (*n* = 99)	Non-Metabolic Bariatric Surgery (*n* = 110)	*p*-Value
Oral iron, *n* (%)				
No	163 (78)	84 (84.8)	79 (71.8)	0.023 ^a^
Yes	46 (22)	15 (15.2)	31 (28.2)	
Vitamin D supplement, *n* (%)				
No	137 (65.6)	61 (61.6)	76 (69.1)	0.256 ^a^
Yes	72 (34.4)	38 (38.4)	34 (30.9)	
Pregnacare supplement, *n* (%)				
No	63 (30.1)	32 (32.3)	31 (28.2)	0.515 ^a^
Yes	146 (69.9)	67 (67.7)	79 (71.8)	
Folic acid supplement, *n* (%)				
No	163 (78)	74 (74.7)	89 (80.9)	0.283 ^a^
Yes	46 (22)	25 (25.3)	21 (19.1)	

^a^ Chi-square test. Groups were compared using chi-square test.

## Data Availability

The dataset used in the analysis of this study is available from the first author upon reasonable request. Contact 201790103@uaeu.ac.ae to request the data used in this study.

## Supplementary Materials

The following supporting information can be downloaded at https://www.mdpi.com/article/10.3390/nu16010072/s1: Table S1: The distribution of the pregnant women with MBS and Non-MBS.
