# Supplementary material for: Micronutrient Status in Pregnant Women after Metabolic Bariatric Surgery in the United Arab Emirates: A Prospective Study
Source: Nutrients. 2023 Dec 25;16(1):72. doi: 10.3390/nu16010072 (PMC10781104; doi:10.3390/nu16010072)
Supplement: Supplementary file 1 [file nutrients-16-00072-s001.zip › nutrients-2758760-supplementary.pdf]

Supplement Material

Table S1: The distribution of the pregnant women with MBS and Non-MBS

Emirates	Percentage	Number
Abu Dhabi	48%	100
Al Ain	15%	32
Dubai	8%	17
Sharjah	14%	29
Ajman	3%	6
Fujairah	10%	21
Ras Al Khaima	2%	4
